# Spatial inequity in access to healthcare facilities at a county level in a developing country: a case study of Deqing County, Zhejiang, China

**DOI:** 10.1186/s12939-015-0195-6

**Published:** 2015-08-19

**Authors:** Cheng Jin, Jianquan Cheng, Yuqi Lu, Zhenfang Huang, Fangdong Cao

**Affiliations:** School of Geographical Sciences, Nanjing Normal University, Nanjing, 210023 P. R China; Jiangsu Center for Collaborative Innovation in Geographical Information Resource Development and Application, Nanjing, 210023 P. R China; Division of Geography and Environmental Management, School of Science and the Environment, Manchester Metropolitan University, Chester Street, Manchester, M1 5GD, UK

## Abstract

**Background:**

The inequities in healthcare services between regions, urban and rural, age groups and diverse income groups have been growing rapidly in China. Equal access to basic medical and healthcare services has been recognized as “a basic right of the people” by Chinese government. Spatial accessibility to healthcare facilities has received huge attention in Chinese case studies but been less studied particularly at a county level due to limited availability of high-resolution spatial data. This study is focused on measuring spatial accessibility to healthcare facilities in Deqing County. The spatial inequity between the urban (town) and rural is assessed and three scenarios are designed and built to examine which scenario is instrumental for better reducing the spatial inequity.

**Methods:**

This study utilizes highway network data, Digital Elevation Model (DEM), location of hospitals and clinics, 2010 census data at the finest level – village committee, residential building footprint and building height. Areal weighting method is used to disaggregate population data from village committee level to residential building cell level. Least cost path analysis is applied to calculate the travel time from each building cell to its closest healthcare facility. Then an integral accessibility will be calculated through weighting the travel time to the closest facility between three levels. The spatial inequity in healthcare accessibility between the town and rural areas is examined based on the coverages of areas and populations. The same method is used to compare three scenarios aimed at reducing such spatial inequity – relocation of hospitals, updates of weighting values, and the combination of both.

**Results:**

50.03 % of residents can reach a county hospital within 15 min by driving, 95.77 % and 100 % within 30 and 60 min respectively. 55.14 % of residents can reach a town hospital within 5 min, 98.04 % and 100 % within 15 and 30 min respectively. 57.86 % of residential building areas can reach a village clinic within 5 min, 92.65 % and 99.22 % within 10 and 15 min. After weighting the travel time between the three-level facilities, 30.87 % of residents can reach a facility within 5 min, 80.46 %% and 99.88 % within 15 and 30 min respectively.

**Conclusions:**

The healthcare accessibility pattern of Deqing County has exhibited spatial inequity between the town and rural areas, with the best accessibility in the capital of the county and poorest in the West of the county. There is a high negative correlation between population ageing and healthcare accessibility. Allocation of more advanced medical and healthcare equipment and highly skillful doctors and nurses to village clinics will be an efficient means of reducing the spatial inequity and further consolidating the national medical security system. GIS (Geographical Information Systems) methods have proven successful method of providing quantitative evidence for policy analysis although the data sets and methods could be further improved.

## Background

Over the past three decades, China has achieved internationally recognized success in its economic development but simultaneously has paid a shocking price in environment protection, resource use and human health improvement. Food security, environmental pollution and health care have become a main concern of Chinese government and people recently [1]. Prior to the economic reform or even before the mid-1980s, China had been one of the most successful countries in providing equal healthcare services [2] as 85 % of the population was able to access to basic health care at a low and affordable cost [3].

The astonishing economic achievements have increased the quantity and quality of healthcare facilities in cities and advanced rural areas markedly [4]. Up to 2011, there had been 954,000 medical centers and healthcare organizations in the entire country [5]. The numbers of professional doctors, qualified nurses, and beds per thousand population have increased from 1.5, 1, and 2.5 in 2002 to 1.8, 1.7 and 3.8 in 2011 respectively [5]. The total expenditure of healthcare, including financial sources from government, social medical insurance, commercial health insurance and residents’ self-funding reached 2434.591 billion yuan RMB in 2011, which is 5.1 % of the country’s GDP [5]. The annual growth rate has increased by 11.32 % based on comparable price since 1978. The total numbers of diagnosed and hospitalized patients have increased from 2.15 billion and 59.91 million in 2002 to 6.27 billion and 150 million in 2011 [5].

However, the current inequities in health care services between regions, urban and rural areas, diverse age groups and income groups have been growing rapidly [2–4, 6–8], due to many political, economic, social and cultural factors (e.g. market commercialization). Though healthcare disparity remains a major public health challenge in many countries [9, 10], e.g. the United States [11], the health inequality in China, may complicate efforts to further development, reduce poverty, and maintain social stability [12] and a disturbing level of inequality in society is particularly observable in the area of health care [4].

Healthcare inequity has multiple dimensions – service delivery, health-care use, health outcomes, health insurance, reimbursement, and access [8, 13, 14]. Among them, equal access to basic medical and healthcare services is particularly important for maintaining social justice, which has been recognized as “a basic right of the people” in the major new policy directions for achieving Healthy China by 2020, launched by the Ministry of Health after the 17th Chinese Communist Party Congress. It is also designated as the principal aim of the rural health insurance reform by the State Council [15]. Access to medical and healthcare services has a variety of dimensions: spatial or physical, financial, social and virtual access, with financial access extensively studied in the published literature (e.g. [12]). The expansion of the insurance program covering 95.7 % population by 2011 seems to have been instrumental in narrowing the inequities in financial access between rural and urban areas [7]. The old Cooperative Medical System (CMS) has been very successful in removing the health inequity during the command economy period but was inefficient after the economic reform due to the marketization of the health sector [12]. A New Cooperative Medical Scheme (NCMS) in rural areas launched in 2003 that aims to safeguard the access of rural people to basic health services and alleviate the financial burden caused by sickness and poverty has contributed to the improvement of financial access in rural China. The evolution of CMS in China is referred to the paper by Liang and Lu [16].

Healthcare accessibility refers to the relative ease by which healthcare resources can be reached from a given location [14, 17, 18], and it is an interface between potential users and healthcare resources [19], such as facilities (hospitals, medical centers and clinics). Therefore, its spatial dimension is highly dependent on the spatial allocation of these facilities. Spatial inequity (or inequity in the spatial accessibility) has been investigated for a variety of groups, services and places (e.g. access of lower-income people to health clinics in city centre [18]). Particularly the disparity between urban and rural areas has been one of main concerns in both developed and developing countries, such as Japan [20] and Costa Rica [21]. Rosenberg proposed an idealist theory to interpret the social justice in access to care [22]. In the meanwhile, there have been inspiring advances in measuring the spatial accessibility due to increasing availability of data sets and improved analytical functions of GIS. Both Wang [14] and Guardiola [17] have provided a recent review of these methodological developments. The popular methods include provider-to-population ratio, distance to nearest provider, average distance to a set of providers and gravitational models of provider influence, kernel density estimation, two-step floating catchment area and space-time model. Although conceptually and methodologically less advanced, travel time or distance to the nearest hospital might be more meaningful, intuitive and transparent for health professionals, policy makers and planners [11, 14].

Spatial accessibility to healthcare facilities has received massive attentions in Chinese case studies, but been less studied particularly at a county level due to limited availability of high-resolution spatial data. For example, Tao et al [23] measured elderly people’s accessibility to residential care facilities in Beijing using census data at sub-district level and optimized the location of these facilities for achieving spatial equity. However, the measurement accuracy could be further improved with high availability of demographic data at a finer scale (e.g. community or residential committee level). Most case studies were focused on city scale due to availability of spatial data including transport network and census data and where spatial inequity between the urban and rural might be different from that on county scale.

This study is focused on measuring spatial accessibility to healthcare facilities on county scale using the census data at village committee level and high-resolution residential building data. The spatial inequity between the urban (town) and rural is assessed and three scenarios are designed and built to examine which scenario is instrumental for better reducing the spatial inequity. Deqing County in the wealthiest Yangtze Delta of China is chosen as a case study.

### Data and methods

#### Study area

**Fig. 1:**
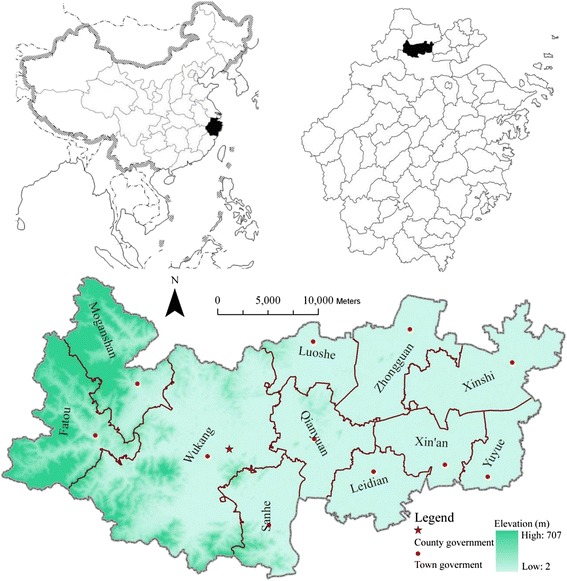
Location of the study area - Deqing County and its towns/townships

Deqing County is situated in the North of coastal province – Zhejiang and in the West of Yangtze Delta (Fig. 1), between latitude 30°261’-30°421’N and longitude 119°451’-120°211’E. Its topography is dominated by hilly areas in the West with the highest elevation value 707 m and low altitude plateau in the East (Fig. 1), with a total area of 935.9 km^2^. This county, as located in a well-developed region, has a total population of 492,000 and local output value of 30.626 billion Yuan RMB in 2012 [24].

The administrative structure in China is a six-level hierarchy: the central government, province/centrally administered city, prefecture, county/district, town/township/sub-district, and village/residential committee (district, sub-district and residential committee in the urban area are the equivalent units of county, town/township and village committee in the rural area respectively). Many healthcare facilities are classified by this administrative structure. For example, county hospital means that it is mostly located at the capital of a county and particularly administered by its county government. County, as a middle-level administrative unit of socio-economic development in China, plays a crucial role in developing medical and healthcare industry. First, the basic medical insurance regulations are operated at county level. Patients can only visit local hospitals within the county area they live in order to reimburse their medical expenses even though a hospital located in a neighboring county is nearer to them. Only when the hospitals in this county are not able to diagnose and treat the patient, the patient can request for a shift to a higher-level (e.g. provincial) hospital with a stamped letter provided by the county hospital. Only a small proportion of actual expenses incurred in the higher-level hospital can be reimbursed based on the maximum limit set in the medical healthcare system. Without the stamped letter from the county hospital, all the expenses have to be paid only by the patients themselves.

Second, a county-level medical healthcare system is a service network composed of county hospital, town/township hospital and village clinic. This three-level network provides daily medical services to a majority of residents in China and accordingly this network is an appropriate representative of the entire national system. The number of healthcare organizations on this network has reached 918, 000, which is 96.23 % of those all over the country [5]. The number of doctors, nurses, beds, medical equipment and operational capacities vary between the three levels (Table 1). Each level plays remarkably varying roles in the entire healthcare system. The county hospital positioned as a head dragon is open to all residents in the county. The village clinic at the lowest level of the system, with only one in each village, is responsible for providing local residents with techniques and advices for precaution, medical treatment, healthcare, health education and family planning.

**Table 1:** Comparisons between county hospital, town/township hospital and village clinic

Indicator	County hospital	Town hospital	Village clinic
Category^a^	More at class 2 and few at class 1	Class 3	Not classified
Number of qualified doctors	>100	40-80	1-2(temporally working doctor allocated by a town hospital)
Number of registered nurses	>100	5-20	0
Number of beds	>200	10-30	0
Equipment	A full set including very expensive one for diagnosis and treatment	No large-size or advanced equipment	No basic ones, only by experiences
Operation	A majority of operations	Only easy-use and low-cost equipment	Not
Deqing County	3	19	135

^a^Hospitals in China are classified into three categories (1-highest, 2-middle and 3-lowest) according to national standard of hospital classification of 1989

**Fig. 2:**
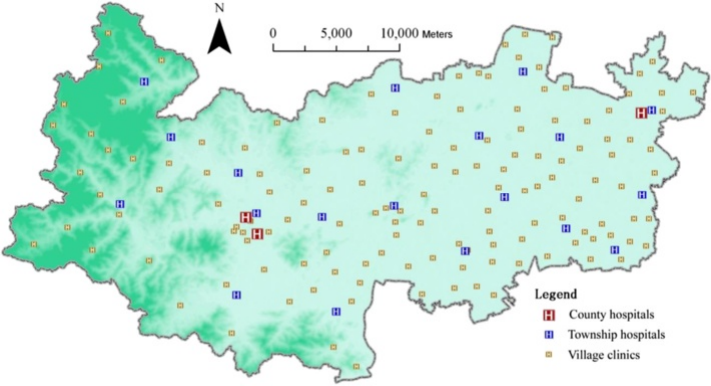
Spatial distribution of all healthcare facilities

Deqing County has 1,060 qualified doctors, 938 registered or charted nurses and 1,796 beds in total by the end of 2013 [25]. Currently, it encompasses 3 county hospitals, 19 town or township hospitals and 135 village clinics (Table 1 and Fig. 2). There is a parallel classification of hospitals: from Class 1 (the highest) to Class 3 (the lowest), based on the resources owned by and the professional skills of doctors and nurses in the hospital (Table 1). The three county hospitals in Class 2 include people’s hospital, Chinese medical hospital and the third people’s hospital. The people’s hospital and Chinese medical hospital are located at Wukang Town, which is the capital of Deqing County. The third people’s hospital is situated in Xinshi Town in the East of the county (Fig. 2). Each town or township has a couple of hospitals meeting the national standard of Class 3 hospital. All the 135 village clinics are assigned with a permanently residing doctor by its town hospital. This three-level public health service system has been formed to serve 492,000 populations in the county.

**Fig. 3:**
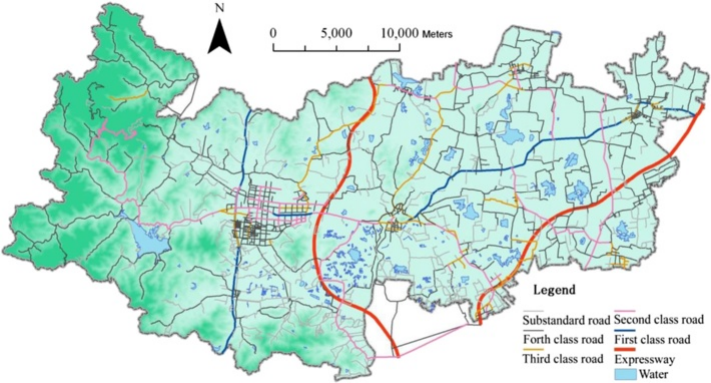
Road network in Deqing County

**Fig. 4:**
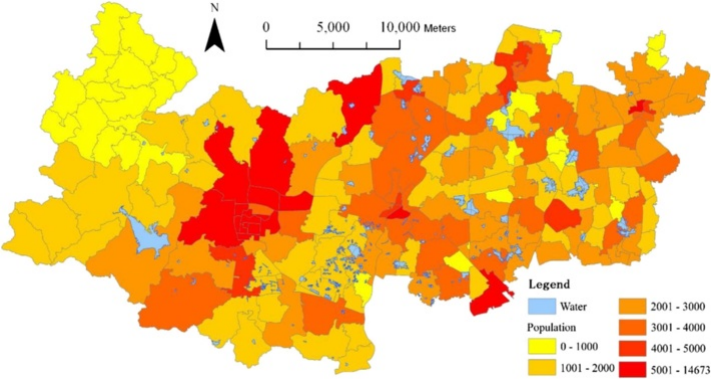
Spatial distribution of population at village committee level

**Fig. 5:**
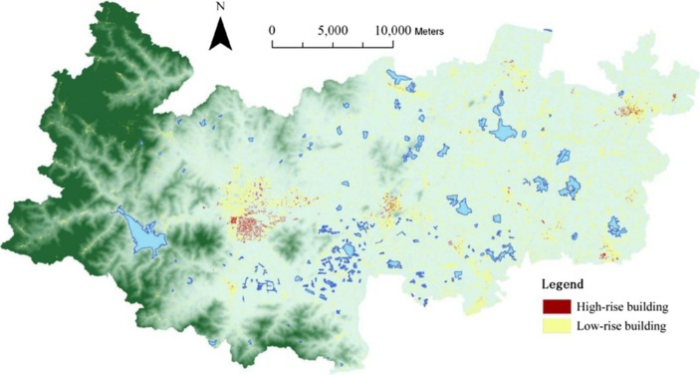
Spatial distribution of residential buildings

The data sets used for this study include DEM (Fig. 1), highway road network (Fig. 3), 2010 population census (Fig. 4), building footprint (Fig. 5), location of all healthcare facilities (Fig. 2). The DEM data has 50 meter resolution. All these data sets are provided by Zhejiang Provincial Centre for Geographic Information System (GIS) for the purpose of government-funded project: national geographical survey (Deqing is one of the first group of experimental sites).

The road network in Deqing is classified as expressway (or motor way), first-class, second-class, third-class, fourth-class and substandard highway (Fig. 3), each of which is assigned with a different car driving speed value (Table 2). Comparatively the west has a lower density of road network due to its hilly terrain.

**Table 2:** Driving and walking speed values on highway networks and land cover

Road class	1^st^	2^nd^	3^rd^	4^th^	substandard	Land cover	Hill
Speed(km/h)	80	60	40	30	20	5	3
Time^a^ (min)	0.0375	0.05	0.075	0.1	0.15	0.6	1

^a^ Time is the minutes travelling through a cell (50 x 50 m) in each road class and land cover

The dominant and reliable source of demographic data is national population census survey. The latest survey was conducted on 1^st^ November 2010, in which the population data can be available for the hierarchy of administrative units: from province, district, sub-district/town/township down to urban community or rural village committee (e.g. [26]). In this study, the total population data at the rural village committee level is collected from the 2010 census shown in Fig. 4. Deqing County has 135 village committees in 2010, among which Wukang town, the capital of this county, has four largest village committees, each with more than 10,000 populations. Conversely, the Western area (Moganshan Town and Fatou Township in Fig. 1) has 16 smaller-size village committees and the smallest one only has 187 populations. A heterogeneous distribution of population across the county has been clearly seen in Fig. 4.

However, the village committee level is spatially too coarse to accurately geo-reference residents and measure the spatial coverage of population who need medical and healthcare services. An ancillary source of GIS data is residential building: footprint and classified height (high-rise – over three floors and low-rise - one or two floors) (see Fig. 5). Due to massive fieldwork, the exact number of floors per building is not available. This is the limitation of this data set.

### Analytical process

The measurement of geographical accessibility to healthcare facilities methodologically can be implemented by using either vector-based network analysis or raster-based least cost path analysis in the published literature [27–29]). Delamater et al. [30] have extensively compared the two methods and concluded that both methods can be applied in different situations. In the rural China, when road network has limited coverage (e.g. the western part in this case study), patients may travel through non-road area by walking, least-cost path analysis would be a suitable method for estimating travel time. The raster-based method allows for any route between locations to be considered, not just those along established roads. The least-cost analysis method reflects the overall mode in which people tend to travel [27]. Cheng et al. [28] used the method to measure and compare the spatial accessibility to residential care resources. They concluded that the least-cost path analysis is able to identify the service areas at certain time thresholds, as well as underserved areas. As a result, in this study, the assessment of spatial inequality in access to healthcare facilities is based on the overall travel time (driving and/or walking) from a residential building cell to each-level closest facilities using the raster-data based least-cost path analysis method.

**Fig. 6:**
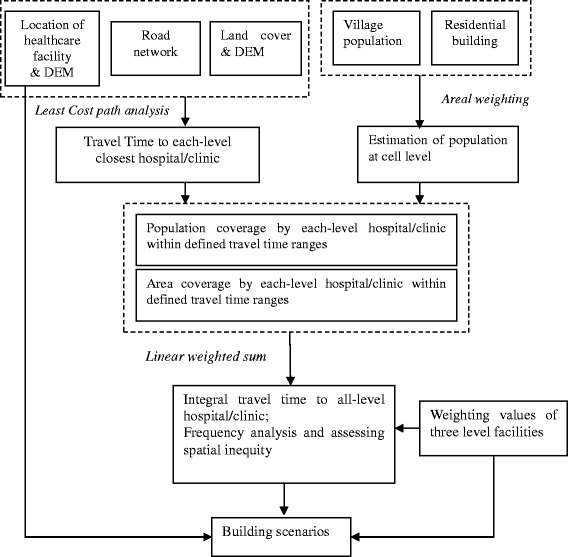
A work flowchart of the methodology

The analysis unit in this paper is a grid of 50 m x 50 m since such a high-resolution grid would improve the accuracy of estimating travel time [30]. This selection is also justified based on the comparative results from the use of 500 m, 100 m, 50 m and 10 m grids respectively. The entire analytical process is represented as a work flowchart shown in Fig. 6. The main methods used include disaggregation of population from village committee level to cell level, calculation of travel time from each residential building cell to the closest facility at each administrative level and the integration of travel time at all three levels.

### Population estimation

To assess the healthcare service, a first step is estimating the population coverage of each facility. The most popular means of achieving this is disaggregating population from a higher-level administrative unit to a lower-level unit (e.g. from output area unit to building areal unit in the UK). The higher resolution the spatial unit has, the more accurate the disaggregation is. The lower-level administrative unit in a county is town or township and the lowest is village committee. The difference between town and township is that the latter has a higher proportion of non-agricultural population. Deqing County has 9 towns, 2 townships and 135 village committees. The average size of village committee is 3644 populations. In this study, the areal weighting method [31] is used to disaggregate population from the village committee level to the residential building cell level.

The population data at the village committee level (Fig. 4) are only assigned to residential building cells, which is rasterized from the polygons of residential buildings in Fig. 5. No population data is assigned to the cells of any other land use or buildings (e.g. commercial buildings). To calculate the weighting values between the high-rise and low-rise residential buildings, 500 high-rise and low-rise residential buildings are randomly sampled across the study area respectively to estimate the average number of floors in each category, which turns out 6.59 floors for high-rise and 2.34 floors for low-rise residential buildings. The following equation (eq.1) is utilized to disaggregate population data from the village committee polygon unit to the building cell unit:

1 $$ {p}_{ij}=\left\{\begin{array}{l}\frac{P_j}{\left(2.34\times {n}_{jl}+6.59\times {n}_{jh}\right)}\times 2.34\\ {}\frac{P_j}{\left(2.34\times {n}_{jl}+6.59\times {n}_{jh}\right)}\times 6.59\end{array}\right. $$

**Fig. 7:**
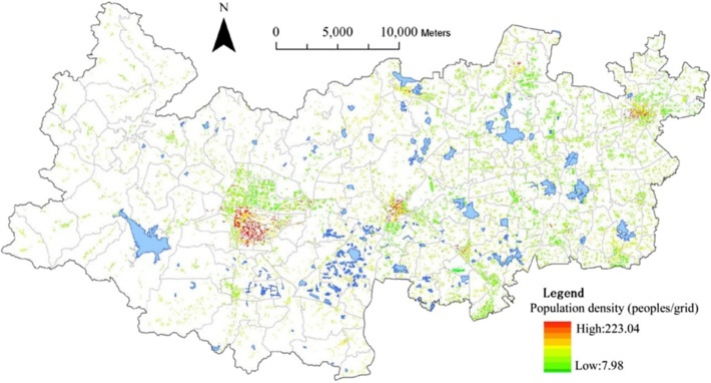
Spatial distribution of estimated population density

Where *P*_*ij*_ is the estimated number of people at cell *i* in village committee *j. P*_*j*_, *n*_*jl*_ and *n*_*jh*_ are the total population, low-rise building cells and high-rise building cells respectively in village committee *j* (see Fig. 4). A total number of 20,733 building cells have a valid value with the minimum 7.98 and maximum 223.04 (i.e. number of people). The areas with the highest population density are located in towns of Wukang, Qianyuan and Xinshi (Fig. 7).

### Least-cost path analysis

As you can see in Fig. 3, Deqing County has a reasonable coverage of highway network and its average car ownership is 0.47 per household in 2012, which is much higher than those less developed regions in China [24], being less than 0.09 per person in 2012 (totally about 120 million automobiles). Hence, driving by car is a prevailing mode of transport for a majority of socio-economic activities, which is chosen as the dominant mode of travel for visiting healthcare facilities. When expressway is used, drivers need to pay for construction and maintenance fees throughout the county. In the most cases of visiting short-distance hospitals, patients would not choose expressway. Consequently, expressway is not included into the least-cost path analysis. The driving speed for each road class (from the first-class to substandard) and its corresponding time travelling through a cell (50 x 50 m) are listed in Table 2 according to local statistics [24]. The land cover classes (mostly artificial areas) are assigned with a same walking speed value of 5 km/h and the hilly terrain area with a walking speed of 3 km/h for simplicity. For some land covers such as water bodies, the travelling speed is set to 0. The DEM data allows the incorporation of slope into the analysis as the topography of terrain may increase or decrease the speed of travel, especially when walking.

The least-cost path analysis is run through the Spatial Analyst extension toolbox in ArcGIS 10.2.2. The travel time from each residential building cell *i* to the nearest healthcare facility at each level *k*: is calculated as T_*ik*_ (K = 1, 2, 3, corresponding to county, township and village) and represented into a raster surface.

The integrated travel time from each residential building cell to three-level healthcare facilities is aggregated into *T*_*i4*_ by the following linear weighted sum formula:

2 $$ Ti4={\displaystyle \sum_{k=1}^n{w}_k{T}_{ik}} $$

Where *T*_*i4*_ is the integral travel time to access to healthcare facilities, *T*_*ik*_ the travel time from building cell *i* to the closest healthcare facility at level *k. n* is 3 (the number of levels). *W*_*k*_ is the weighting value allocated to healthcare facility at level *k*, alternatively interpreted as the relative frequency of visiting the facilities at this level. *W*_*k*_ is calculated as follows:

3 $$ Wk=\frac{Vk}{{\displaystyle \sum_{s=1}^3Vs}} $$

Where *V*_*k*_ and *V*_*s*_ are the total number of visitors to the facilities at level *k* and *s* respectively.

### Catchment area analysis

Catchment area analysis aims to calculate the accumulated areas A_k_ (T_0_) or residents R_k_ (T0), which can be accessed from its closest healthcare facility at each level *k* (1 ~ 4, corresponding to county, town, village and integral levels) within a user-defined threshold time *T*_*0*_. A_k_ (T_0_) and R_k_ (T_0_) are calculated as follows (eq. 4 and 5):

4 $$ Ak(T0)={\displaystyle \sum_{i=1}^n AREAi}\ \mathrm{if}\kern0.5em {\mathrm{T}}_{\mathrm{ik}}\kern0.5em <\mathrm{T}0 $$

5 $$ Rk(T0)={\displaystyle \sum_{i=1}^n Pi}\kern0.5em \mathrm{if}\ {\mathrm{T}}_{\mathrm{ik}} < {\mathrm{T}}_0 $$

In equations 4 and 5, AREA*i* is the area of cell (2500 m^2^ in this case) at location *i. P*_*i*_ is the number of residents at location *i* that was calculated from equation 1 (Fig. 7). *n* is the total number of residential building cells. Equations 4 and 5 are implemented using the Raster calculator tool in ArcGIS 10.2.2. For the purpose of visualization and interpretation, *T*_*0*_ can have a variety of intervals (from 2.5 to 15 min). Mapping *T*_*ik*_ and *C*_*i*_ enables us to detect any spatial inequity in healthcare access across the county. A_k_ (T_0_) and R_k_ (T0) are presented as (accumulative) histograms for statistical comparisons between the four levels (k).

## Results

**Fig. 8:**
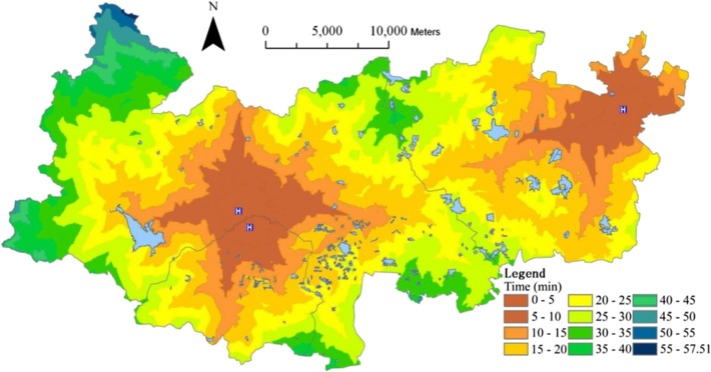
Travel time to the nearest county hospital

Based on *T*_*i1*_, the travel time to the nearest county hospital is mapped with an interval of 15 min into Fig. 8, which reveals a significant spatial variation across the county. Town areas have much shorter travel time than rural areas.

Based on *A*_*1*_ (*T*_*0*_), the total building areas being able to access to the county hospitals within the varied threshold time *T*_*0*_ are statistically summarized into a histogram (Appendix 1a). The graph shows that 27.02 % of residential building areas can reach a county hospital within 15 min, 85.38 % and 100 % within 30 and 60 min respectively. Only 1.43 % areas need more than 45 min for the service.

Based on *R*_*1*_ (*T*_*0*_), the total number of residents being able to access the county hospitals within the varied threshold time *T*_*0*_ are statistically summarized into a histogram (Appendix 1b), which shows that 50.03 % of residents can reach a county hospital within 15 min, 95.77 % and 100 % within 30 and 60 min respectively. Even 29.15 % of residents can reach a county hospital within 5 min. Only 0.20 % needs more than 45 min for the service.

Based on *T*_*i2*_, the travel time to the nearest township hospital is mapped with an interval of 5 min into Appendix 2, exhibiting a significant spatial variation across the county, with the longest travel areas – 32.90 min located in the Western Fatou Township.

Based on *A*_*2*_ (*T*_*0*_), the total building areas being able to access the township hospitals within the varied threshold time *T*_*0*_ are statistically summarized into a histogram (Appendix 3a). The graph shows that 86.04 % of residential building areas can reach a township hospital within 15 min, 99.91 % within 30 min except the small areas located in the western Fatou Township. Only 1.04 % areas need more than 20 min for the service.

Based on *R*_*2*_ (*T*_*0*_), the total number of residents being able to access the township hospitals within the varied threshold time *T*_*0*_ are statistically summarized into a histogram (Appendix 3b), which shows that 55.14 % of residents can reach a town hospital within 5 min, 98.04 % and 100 % within 15 and 30 min respectively. 29.15 % of residents can reach a hospital within 5 min. Less than 2 % need more than 15 min for the service.

Based on *T*_*i3*_, the travel time to the nearest village clinic is mapped with an interval of 2.5 min into Appendix 4. It can be clearly seen that the East side has better service than the West, and with a majority of areas covered by 15 min.

Based on *A*_*3*_ (*T*_*0*_), the total building areas being able to access the village clinics within the varied threshold time *T*_*0*_ are statistically summarized into a histogram (Appendix 5a), showing that 57.86 % of residential building areas can reach a village clinic within 5 min, 92.65 % and 99.22 % within 10 and 15 min respectively except the small areas located in Southern Sanhe Township. Only 2.65 % need more than 10 min for the service.

Based on *R*_*3*_ (*T*_*0*_), the total number of residents being able to access the village clinics within the varied threshold time *T*_*0*_ are statistically summarized into a histogram (Appendix 5b), showing that 88.75 % of residents can reach a village clinic within 5 min, 99.56%and 99.97 % within 10 and 15 min respectively.

**Fig. 9:**
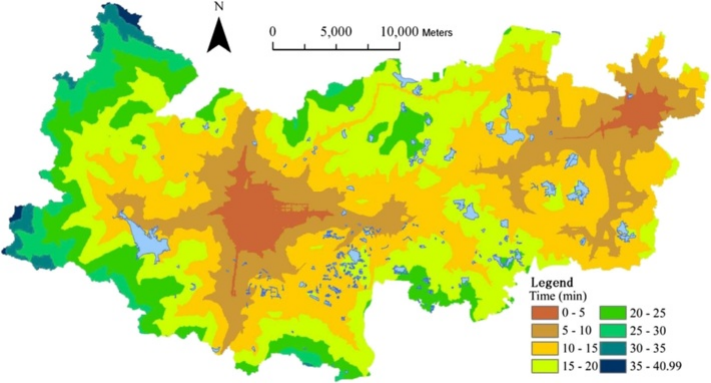
Travel time to the health facilities at all levels

Based on the total number of patients who visited the healthcare facilities at all levels in 2013 [25], the weighting values between the three-level facilities *W*_*k*_ are calculated into Table 3 according to equation 3. The integral travel time to the closest facilities is also calculated using equation 4 and its spatial distribution is mapped into Fig. 9. Two towns (Wukang and Xinshi) that encompass three county hospitals demonstrate the greatest access to healthcare facilities. Conversely, the western area, which is far away from the county hospitals and has sparse highway network and tough terrain, has the lowest level of access, and with the longest travel time - 40.99 min.

**Table 3:** Weighting values of healthcare facilities between the three levels

	County hospital	Town/township hospital	Village clinic
Total number of visitors	2164520	1703132	421045
Weighting values (W_*k*_)	50.47 %	39.71 %	9.82 %

Based on *A*_*4*_ (*T*_*0*_), the total building areas being able to access all the facilities within the varied threshold time *T*_*0*_ are statistically summarized into a histogram (Appendix 6a). The graph shows that 4.302 % of residential building areas can reach a facility within 5 min, but 55.52 % and 98.18 % within 15 and 30 min respectively except the small areas located in Southern Sanhe Township. Only 1.82 % areas need more than 30 min for the service.

Based on *R*_*4*_ (*T*_*0*_), the total number of residents being able to access all the facilities within the varied threshold time *T*_*0*_ are statistically summarized into a histogram (Appendix 6b), showing that 30.87 % of residents can reach a facility within 5 min, 80.46 %% and 99.88 % within 15 and 30 min respectively. Only 0.12 % residents need more than 30 min for the service.

All these analyses mentioned above indicate the presence of a certain degree of spatial inequity in the access to healthcare facilities particularly between the urban and rural areas.

## Discussions

Reducing inequities in access to basic health services requires a more generic and comprehensive approach to organizing the healthcare system [27]. The analyses mentioned above indicate a generally good access to healthcare facilities but which exhibits a spatial inequity between the urban and rural areas across the county. Two urban areas (towns in this case): Wukang (the capital of this county) and Xinshi (the second center in the east) are able to reach the facilities within 5 min. Conversely, the hilly areas in the West have the poorest access. This spatial inequity is dominantly caused by the imbalanced social development strategy between the town and rural areas.

First, the higher-level hospitals with high availability of more advanced equipment are all located in the centers of county, though they are committed to serving all the residents in the county. Secondly, both the number of doctors with professional skills and the quantity of costly equipment and instruments in village clinics far lag behind those in town hospitals, letting alone county hospitals. This disparity has worsened the spatial inequity, as rural patients have to travel further for treating their critical illnesses that cannot be treated in village clinics and even town hospitals. As a result, it is very imperative for local government (e.g. county) to adjust their social development strategy.

### Population ageing

The health of elderly people particularly in rural China has received growing attention [16]. The elderly population at the age of 60 or over in China have reached 178 million in 2010, accounting for 13.3 % of its total population [23], much higher than the international standard of population ageing society, 10 %. The proportion of elderly people at the age of 60 or above over the total population has reached 16.2 % in Deqing County, far more than 13.8 % at the province level and 13.3 % at the national level, based on the sixth national population census of 2010. The same proportion of population ageing at town/township level as shown in Appendix 7 exhibits the minimum 10.71 % and maximum 26.80 %. It indicates all towns and townships are an ageing society. However, the most ageing town is Fatou Township and Moganshan Town in the West, with 26.80 % and 26.56 % respectively, contrasting with the least ageing town – Wukang Town with 10.71 %. There are several factors contributing to the pattern of population ageing at county level. Firstly, majorities of young people in rural areas have migrated to the capital of this county – Wukang Town for working and living as it is the political, economic, and cultural center of the county. This has led to massive loss of labors in the rural area. Secondly, the senior people have less preference to live in town or city because they are not able to find suitable jobs as well as to adapt to urban life. All of these have exerted much higher pressure on the western area where its economic level is relatively very low due to hilly terrain.

Obviously, a challenge for population ageing is to improve elderly people’s access to healthcare facilities. Senior people’s frequency of visiting hospitals is much higher than that of young people, which results in their increasing demand for better access. However, in this case study, there is a significant imbalance or negative correlation between the spatial distributions of ageing population and healthcare accessibility. The western area (Moganshan Town and Fatou Township) with the highest degree of population ageing has the poorest healthcare accessibility but the capital of county – Wukang town with the lowest degree of population ageing has the best healthcare accessibility. This imbalance has been prevailing at county level across China: county capitals have accumulated all the best medical resources but they are less accessible to large groups of senior people in remote areas. This has become the main concerns of central and local governments with basic medical and healthcare system development.

### Scenarios for improving the accessibility

The potential solutions to the imbalance issues might be twofold: optimal allocation of healthcare facilities and adjustment of weighting values between the three levels. For the former solution – optimal allocation, there is little space to re-allocate healthcare facilities at town and village levels as these facilities have been evenly distributed across the study area. However, it is possible to optimize the locations of three county hospitals. The county government has accepted a proposal to relocate the Chinese medical hospital to Qianyuan Town in the central so that Qianyuan and Leidian towns will have improved coverage of populations. Alternatively, the weighting values between the three-level facilities can be updated to cover further rural areas. Comparatively, the village clinics that have good spatial coverage are less attractive to patients than hospitals at higher levels due to their poor skills of medical healthcare and insufficient provision of advanced equipment. Consequently, improving the healthcare level of village clinics may increase the patients’ frequency of visiting facilities at this level.

Three scenarios are proposed based on the rationale mentioned above. The first one is only changing the allocation of healthcare facilities but not updating their weighting values. The second is a reverse of the first, i.e. not changing the allocation but updating the weighting values. The third is the combination of both: not only changing the allocation of hospitals but also updating their weighting values. The travel time to the closest healthcare facility in response to each scenario are re-calculated and their comparisons are listed into Table 4.

**Table 4:** Comparisons between the three scenarios

Items	Scenario one	Two	Three
Maximum(min)	40.99	33.58	33.58
Average(min)	14.03	11.73	11.28
Proportion of improved areas(%)	29.68	98.89	98.99
Proportion of declined areas(%)	10.90	1.11	1.01
Proportion of no-change areas(%)	59.42	0	0
Maximum declined(min)	4.31	8.87	8.87
Maximum increased(min)	1.91	0.05	1.31
Average improved(min)	0.68	2.98	3.43

For the first scenario, the Chinese medical hospital is relocated from Wukang (Fig. 2) Town in the central west to Qianyuan Town in the central (Appendix 8). The travel time from each residential building cell to its closest county hospital is recalculated and represented into Appendix 8, which demonstrates better spatial coverage than the previous one in Fig. 8.

**Fig. 10:**
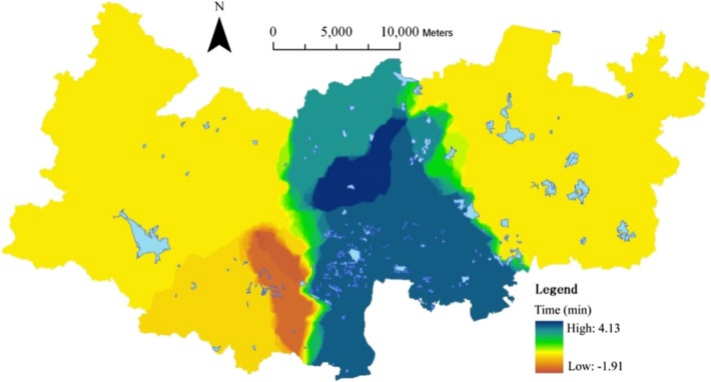
The updated travel time in scenario One

Using the same weighting values (50.47 %, 39.71 % and 9.82 %) as before (Table 4), the travel time to the healthcare facilities at three levels (Appendix 2, 4 and 8) are combined according to equation 2. The integral accessibility is updated into Appendix 9. Fig. 10 (= Appendix 9 – Fig. 9) shows the difference in the level of accessibility between scenario one and the current one.

Figure 10 exhibits that the average travel time in scenario one is only reduced by 0.68 min. 29.68 % of cells have improved their accessibility through reducing their travel time by 4.13 min at most. 59.42 % of cells have no change. 10.90 % areas have increased their travel time by 1.91 min at most. Generally, there is no significant change in scenario one except the central area.

**Fig. 11:**
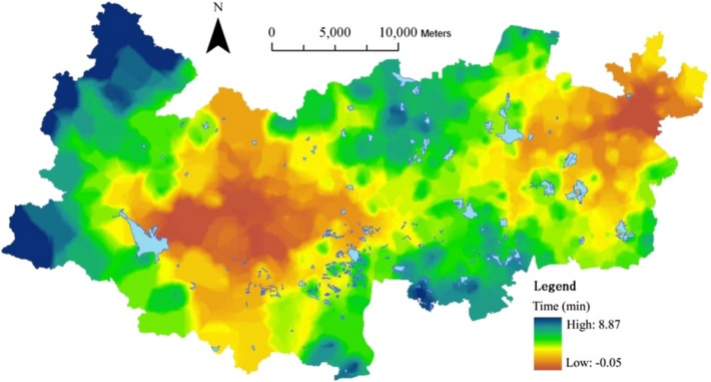
The updated travel time in scenario Two

In scenario Two, only the weighting values of healthcare facilities between the three levels are updated as one third, i.e. an equal value for each level. Then the integral accessibility is updated and represented into Appendix 10. Its difference from the current one is represented into Fig. 11, which exhibits that the average travel time in scenario two (11.73 min) has been reduced by 2.98 min. 98.89 % of cells have improved their accessibility through reducing their travel time by 8.87 min at most. Only 1.11 % of building cells have increased their travel time by 0.05 min at most. Generally, there is significant change in scenario two particularly in the west.

**Fig. 12:**
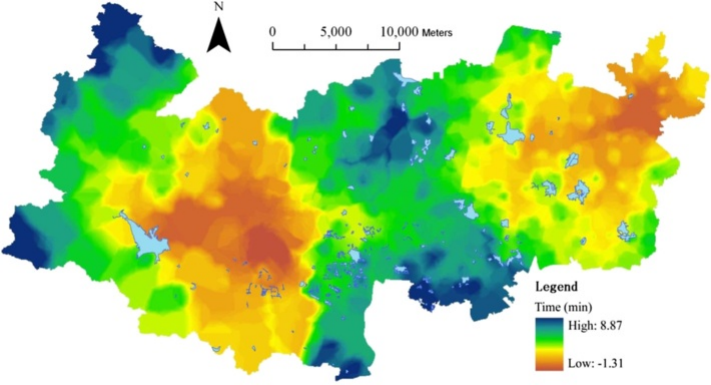
The updated travel time in scenario Three

Scenario Three is a combination of scenarios one and two, meaning the relocation of the third county hospital and updates of weighting values between the three levels. The integral accessibility is updated and represented into Appendix 11. Its difference from the current level is represented into Fig. 12, demonstrating that the average travel time in scenario Three (11.28 min) has been reduced by 3.43 min. 98.99 % of building cells have improved their accessibility through reducing their travel time by 8.87 min at most. Only 1.01 % of cells have increased their travel time by 1.13 min at most. Generally, there is very significant change in scenario three, not only the western but also the central areas.

The outcomes of three scenarios demonstrate the impacts of updating the weighting vales of healthcare facilities between the three levels on healthcare accessibility particularly in the economically poor western part (in scenarios Two and Three). The average travel time across the county can be reduced to about 11 min. This means improving the healthcare levels of village clinics are crucial for reducing the spatial inequality in healthcare accessibility between the urban and rural, and between the young and senior people because the main healthcare resources are in cities and towns. This is particularly important for rural area that has a higher degree of population ageing than urban area and is increasingly demanding social justice. There are 5.8 doctors and 5.8 nurses for every 1000 residents in large cities, but only 0.6 doctors and 0.4 nurses per 1000 population in the less developed rural areas [4]. As the largest developing county, the social justice issue in China is largely caused by the imbalanced allocation of social resources – such as medical and healthcare resources in this case. The question of how to urbanize rural residents is a great challenge for urbanization at this new stage in China. The medical systems during Chairman Mao’s period have benefited rural people as more skillful doctors (called ‘barefoot’ doctors) were sent over to village clinics or medical centers [3, 12, 16]. The general physician (GP) system in the western countries such as UK might be an adoptable model for improving the village clinics in China. It means the family physicians at a small-scale medical center available in each village should be the first doctor to diagnose all the patients in the rural area and only some of them will be recommended to county hospitals. Through such a process, both patients’ healthcare accessibility and county hospital’s pressure on hosting a huge number of patients can be improved.

## Conclusions

Taking Deqing County as a case study, this paper has measured the travel time from each residence place to its closest healthcare facility at three different levels and evaluated the integral accessibility to healthcare services in the county. First, Deqing County has a generally reasonable provision of healthcare services as 80.46 %% and 99.88 % of population can reach a facility within 15 min and 30 min respectively. However, the accessibility pattern has exhibited spatial inequity between the town and rural areas, with the best in the capital of county and poorest in the West. Second, the simulation results from three built scenarios have confirmed that an increase of weighting value of village clinics has significantly reduced the spatial inequity between the urban and rural areas. The average travel time throughout the county can be remarkably reduced to 11.73 min. This has implied that allocation of more advanced medical and healthcare equipment and highly skillful doctors and nurses into village clinics is a very important step to maintain and consolidate the national medical security system.

In terms of spatial accessibility, this paper is only focused on location of healthcare facilities and the method of aggregate measurement. In the future, other aspects of healthcare accessibility – health needs, healthcare availability (e.g. facility capacity), spatial competitions (between patients and between facilities), spatial diversity (e.g. medical services and treatments) should be incorporated into the measurement, which can be disaggregated by age, gender, and health status when the relevant data sets become available (see an example in job accessibility by Cheng and Bertolini [32]). The vector data based network analysis method may calculate the travel time more accurately for urban and town areas as complicated road network (e.g. overpass and underpass) and detailed traffic rules (e.g. one-way and turn restrictions) can be considered to represent a multiple-mode transport network. It will be interesting to compare the strengths and weaknesses of the two methods applied to a rural county. In this paper, scenario building was simulated by updating three weighting values and subjective allocation of county hospitals. Further consideration of many other factors (such as population composition, inpatient-outpatient ratio, and emergency cases) into the calculation of weighting values may make the scenario analysis be linked with local policy practice more tightly. Using more data sets about healthcare facilities, a spatial optimization model should be developed to maximize equal accessibility between the town and rural areas in the future (e.g. Tao, et al., [23]). GIS has been proven a successful method of providing quantitative evidences for policy analysis though data sets and methods could be further improved.

Also in China, the largest developing country, self-payment for treatment (particularly operations) in hospitals is still a heavy burden for a majority of patients in rural areas. As a result, not only spatial accessibility but also social and financial accessibility should be one of the main concerns by central and local governments. In the less developed rural areas, people in general have a lower level of education and poor access to information about health [4]. Therefore, virtual accessibility to internet and other health resources also exhibit a certain level of inequity. The current analysis assumes that accessibility is gender and age neutral; this may not be the case in a county of China. Gender- and age-based inequalities in driving limit the women’s, children’ and elderly people’ ability to access to the healthcare they need, as they all may not have predominant access to cars in rural China.

## Figures and Tables

**Fig 13:**
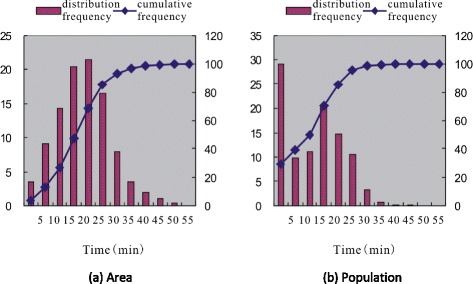
Statistical pattern of spatial coverage by county hospitals

**Fig 14:**
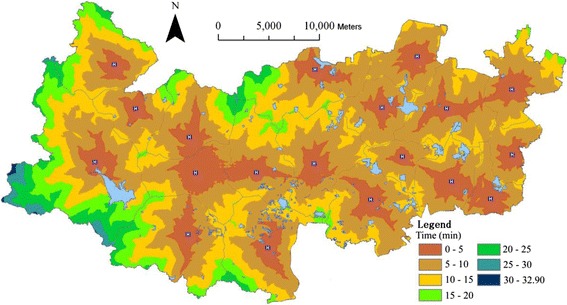
Travel time to the nearest township hospital

**Fig 15:**
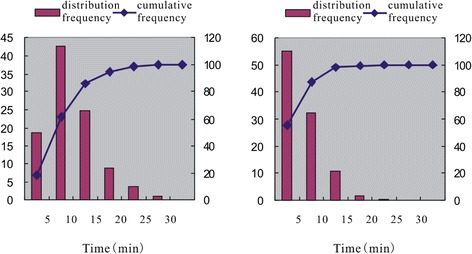
Statistical pattern of spatial coverage by township hospitals

**Fig 16:**
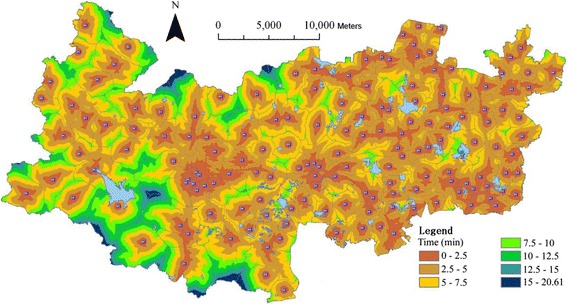
Travel time to the nearest village clinic

**Fig 17:**
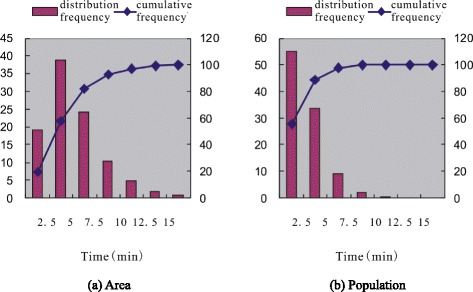
Statistical pattern of spatial coverage by village clinics

**Fig 18:**
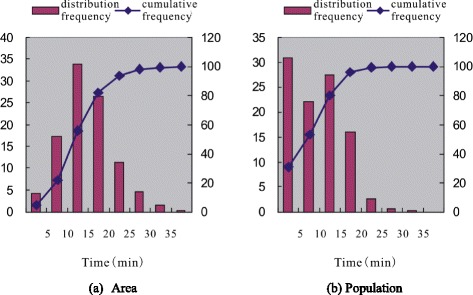
Statistical pattern of spatial coverage by all facilities

**Fig 19:**
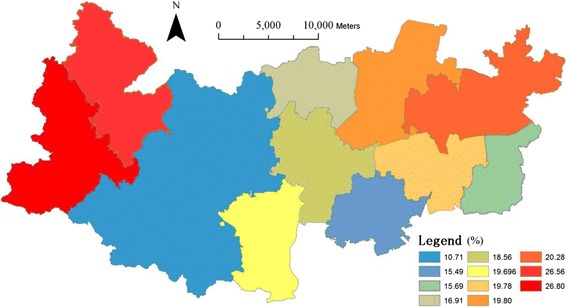
Spatial distribution of population ageing

**Fig 20:**
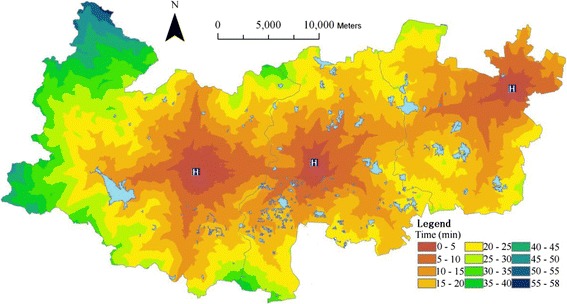
Recalculated travel time to the closest county hospital

**Fig 21:**
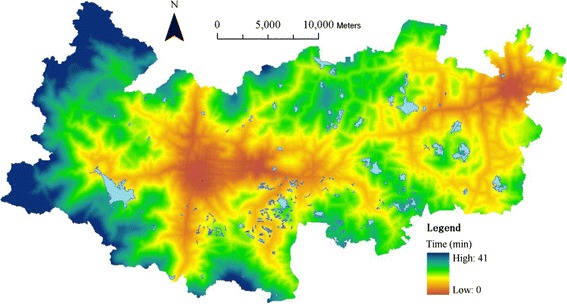
Integrated travel time to all health care facilities in response to scenario one

**Fig 22:**
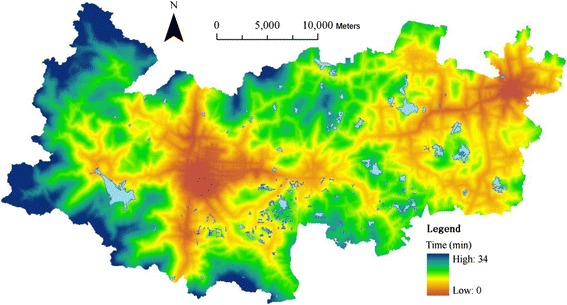
Integrated travel time to all the healthcare facilities in scenario two

**Fig 23:**
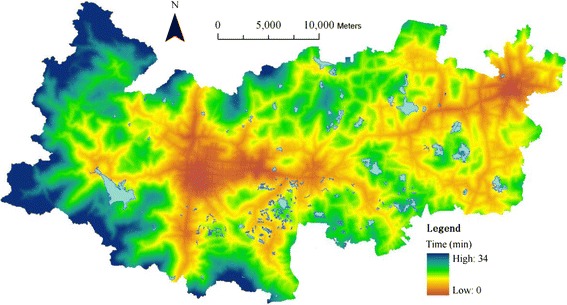
Integrated travel time to all the healthcare facilities in scenario three
